# Plant systemic induced responses mediate interactions between root parasitic nematodes and aboveground herbivorous insects

**DOI:** 10.3389/fpls.2013.00087

**Published:** 2013-04-12

**Authors:** Mesfin Wondafrash, Nicole M. Van Dam, Tom O. G. Tytgat

**Affiliations:** ^1^Department of Ecogenomics, Institute for Water and Wetland ResearchRadboud University Nijmegen, Nijmegen, Netherlands; ^2^School of Plant Sciences, Haramaya UniversityDire Dawa, Ethiopia

**Keywords:** aboveground–belowground interactions, signaling interactions, systemic induced plant defense, secondary plant compounds, herbivores

## Abstract

Insects and nematodes are the most diverse and abundant groups of multicellular animals feeding on plants on either side of the soil–air interface. Several herbivore-induced responses are systemic, and hence can influence the preference and performance of organisms in other plant organs. Recent studies show that plants mediate interactions between belowground plant parasitic nematodes (PPNs) and aboveground herbivorous insects. Based on the knowledge of plant responses to pathogens, we review the emerging insights on plant systemic responses against root-feeding nematodes and shoot-feeding insects. We discuss the potential mechanisms of plant-mediated indirect interactions between both groups of organisms and point to gaps in our knowledge. Root-feeding nematodes can positively or negatively affect shoot herbivorous insects, and *vice versa*. The outcomes of the interactions between these spatially separated herbivore communities appear to be influenced by the feeding strategy of the nematodes and the insects, as well as by host plant susceptibility to both herbivores. The potential mechanisms for these interactions include systemic induced plant defense, interference with the translocation and dynamics of locally induced secondary metabolites, and reallocation of plant nutritional reserves. During evolution, PPNs as well as herbivorous insects have acquired effectors that modify plant defense responses and resource allocation patterns to their advantage. However, it is also known that plants under herbivore attack change the allocation of their resources, e.g., for compensatory growth responses, which may affect the performance of other organisms feeding on the plant. Studying the chemical and molecular basis of these interactions will reveal the molecular mechanisms that are involved. Moreover, it will lead to a better understanding of the ecological relevance of aboveground–belowground interactions, as well as support the development of sustainable pest management technologies.

## INTRODUCTION

Under natural conditions plants are constantly exposed to various herbivorous organisms feeding on above- and belowground parts. The influence of root-feeders on shoot defense and the patterns of aboveground herbivory and *vice versa* remained unrecognized for a long time (Kaplan et al., 2008a). Most of the earlier knowledge on plant–herbivore interactions emanated from studies conducted on leaf herbivory alone, thereby neglecting plant-mediated interactions between the two herbivore communities (Johnson et al., 2006). However, during the last decade, studies on the interactions between these two spatially separated communities, below- and aboveground herbivores, have substantially increased after scientists began to realize that host plants are serving as mediators of these interactions. The outcomes of such studies witnessed that these herbivore communities rarely function independently, but rather interact continuously with each other via their host plants (Bezemer and van Dam, 2005; Kaplan et al., 2009). Belowground feeding organisms such as insects, nematodes, root pathogens, and ectomycorrhizal fungi are known to influence the concentration of plant defense compounds such as terpenoids, glucosinolates, or phenolics, both in the roots as well as in aboveground plant tissues (Manninen et al., 1998; Bezemer et al., 2004; Kaplan et al., 2009; van Dam, 2009).

Plant parasitic nematodes (PPNs) are so abundant and diverse, that plants almost always interact with them during their lifetime (Sohlenius, 1980). Recent studies have shown that due to their omnipresence, PPN are a key driving force of plant succession in natural environments (De Deyn et al., 2003). They also pose a significant threat to global food production, with annual crop losses due to PPN estimated to be more than a 100 billion US$ (Chitwood, 2003). Similarly, about half of all insect species feed on plants (Schoonhoven et al., 2005). With only a few exceptions, PPN are root-feeders, while the majority of insects feed on aboveground plant parts, which have a higher nutritive quality than roots (Hunter, 2001; van Dam, 2009). Therefore, both groups of herbivores are very suitable to investigate the mechanisms of plant-mediated above–below ground interactions. Recently, the first studies were performed that analyzed the interactions of PPN and insects (Wardle et al., 2004; van Dam et al., 2005; De Deyn et al., 2007; Wurst and van der Putten, 2007; Kaplan et al., 2008a, 2009; Olson et al., 2008; Lohmann et al., 2009; Hong et al., 2010; Vandegehuchte et al., 2010). It seems that the outcome of the interaction between both groups of plant feeders can either be negative or positive. However, the knowledge on the occurrence of interactions between these spatially separated herbivore communities remains scattered and poorly documented. Therefore, in this review we discuss the current knowledge on the plant defense against PPN and herbivorous insects, present some examples of the plant-mediated interactions between both groups, indicate the gaps in knowledge and finally identify future research directions.

## FUNCTIONAL DIVERSITY OF NEMATODES AND INSECTS

It has been suggested that the feeding habit (functional guild) of herbivorous insects and plant-parasitic nematodes involved may be one of the factors that determine the outcome of plant-mediate insect–nematode interactions (Mateille, 1994; van Dam et al., 2003, 2005; Bezemer and van Dam, 2005; Wurst and van der Putten, 2007). Therefore, it is important to discuss the diversity in feeding habits of both groups of herbivores and the specific process involved in each feeding habit.

### NEMATODES

Although the basic body plan of all nematodes is highly similar, the genetic diversity is enormous and reflects the long evolutionary trajectory of the phylum (Blaxter, 1998). Phylogenetic analysis revealed that within the Nematoda, plant parasitism evolved at least three times (Blaxter et al., 1998). However, all PPN have common features that arose by convergent evolution to adapt to plant parasitism (Hussey, 1989). They all possess a hollow protrusible stylet that is used to puncture cell walls, inject secretions, and ingest nutrients from the plant cell. The stylet secretions are synthesized in unicellular pharyngeal glands that are much more developed in PPN than in free living nematodes. According to their feeding habit, PPN can be classified into ectoparasites, migratory endoparasites, and sedentary endoparasites (Sijmons et al., 1994; Tytgat et al., 2000).

Ectoparasitic nematodes do not enter the host tissues with their body, but rather puncture plant cells using their stylet and feed on the content of the cells. Depending on the species, feeding can prolong for a few hours till several days. The size of the stylet determines where and how the nematodes in this group feed. Ectoparasitic nematodes with a short stylet (e.g., Trichodoridae, *Tylenchorhynchus* spp.) often feed on root hairs and epidermal cells, while those with a long stylet (e.g., Longidoridae, *Belonolaimus*, *Helicotylenchus *spp.) feed on cortical or even endodermal cells. They insert their stylet into the host cell, inject glandular secretions that dissolve the cell content, and ingest the cytoplasmic contents. Depending on the species, these actions lead to wounding, extensive necrosis, or even gall formation of the root tissue (Sijmons et al., 1994).

Migratory endoparasites are equipped with a robust stylet, which renders them the ability to penetrate and continuously migrate through the root while feeding on the cytoplasm of cortical cells (Sijmons et al., 1994). With the exception of some shoot parasites (Anguinidae and Aphelenchoididae), they all belong to the family of Pratylenchidae (e.g., *Pratylenchus *spp., *Radophulus *spp.). Migration inside the roots is aided by the release of cell wall degrading enzymes via the stylet (Haegeman et al., 2012). Extensive necrosis and sometimes galling or swelling of the root tissue are typical symptoms that develop as a result of infection with such nematodes.

Sedentary endoparasitic nematodes have the most evolved interactions with their host. After root penetration and migration, they induce permanent feeding cells inside the vascular cylinder. The best studied are the cyst nematodes and root-knot nematodes (Hussey, 1989; Davis and Mitchum, 2005). Freshly hatched juveniles penetrate the roots close to the root tip, and migrate intracellularly (cyst nematodes) or intercellularly (root-knot nematodes) toward the vascular cylinder. Also here, migration is performed by vigorous stylet thrusting and secretion of a mix of cell wall degrading enzymes (Davis et al., 2008; Haegeman et al., 2012). After arrival at the vascular cylinder, they puncture the cell wall of a certain cell and start repeated cycles of stylet secretion release into the cytoplasm and ingestion of cytoplasmic content (Wyss, 2002). The initial feeding cell responds with an extensive change in gene expression and morphology (Gheysen and Fenoll, 2002; Caillaud et al., 2008). The cells become hypertrophic and show a huge proliferation of all organelles. Root-knot nematodes induce six to seven giant cells, which become multinucleated by repeated mitosis without cytokinesis. In contrast, cyst nematodes induce a syncytium, which is formed by cell wall dissolution of the initial feeding cell and fusion with the neighboring cells. In clear contrast to the migratory endoparasitic and ectoparasitic nematodes that mostly kill the cells on which they feed, the sedentary endoparasitic nematodes maintain their feeding cells healthy and metabolically active throughout their life cycle. Once they started feeding, they even lose their locomotory muscles, and become completely depended on the hypertrophic cells for further development. Though the giant cells and the syncytia are distinct in their development, functionally they are similar in that they serve as transfer cells of nutrients derived from the phloem (Offler et al., 2003; Hoth et al., 2005, 2008). While cyst nematodes species show specificity for certain plant families, root-knot nematodes such as *Meloidogyne incognita *for instance have an extremely wide host range comprising almost all families of flowering plants (Trudgill and Blok, 2001).

### INSECTS

Based on their feeding habits herbivorous insects are classified into leaf chewing, mining and boring, sap-sucking, gall inducing, and seed predating (Schoonhoven et al., 2005; Gullan and Cranston, 2010). The majority of leaf chewing insects belong to the family Lepidoptera, Coleoptera, Orthoptera, and Hymenoptera. Different developmental stages, such as the caterpillars of lepidopterous (moths and butterflies), the larvae and adults (beetles) of coleopterous insects and the nymphs and adults of orthopterous insects feed on the leaves of plants. Other plant parts such as roots, shoots, stems, flowers, or fruits are also eaten by this group of insects (Schoonhoven et al., 2005; Gullan and Cranston, 2010).

Larvae of plant mining and boring insects live and feed on the internal tissues of plants. For instance, leaf mining insects live and feed between the two epidermal layers of a leaf and ultimately they leave behind a thin layer of dry epidermis (Connor and Taverner, 1997; Sinclair and Hughes, 2010). The damage due to this group of insects often appears as tunnels, blisters, or blotches on the leaf. This leaf mining habit is confined only to insects belonging to the orders Diptera, Lepidoptera, Coleoptera, and Hymenoptera (Gullan and Cranston, 2010). Plant boring insects exhibit a broad range of feeding habits, which can be categorized based on the plant part they damage. These could be stalk borers that attack grasses and more succulent plants; wood borers feeding on twigs, stems, and/or trunks of woody plants; borers that damage roots and belowground plant storage organs such as tubers, corms, and bulbs; fruit borers that destroy or reduce the reproductive output of many plants because the larvae consume the fruit tissues (Schoonhoven et al., 2005; Gullan and Cranston, 2010).

Typical for the sap-sucking insects are the modified mouth parts that are fused to form a stylet (Labandeira, 1997). The stylet is used to penetrate the host tissue, to inject saliva into the host tissue and to retrieve sap from the plant. Depending on the species, the stylet may penetrate superficially into a leaf or deep into the plant tissue, following either intra- or intercellular paths (Kaloshian and Walling, 2005). Because a different reaction toward systemic plant responses can be expected, a functional distinction should be made between cell content and phloem or xylem feeders. Many Heteroptera and thrips feed on the cell content of epidermal or parenchymal cells (Heming, 1993; Schoonhoven et al., 2005). Phloem feeding is performed by most aphids, mealy bugs, soft scales, psyllids, and leafhoppers, while spittle bugs and cicadas feed on xylem sap (Gullan and Cranston, 2010). Because only the stylet penetrates the plant tissue, sap-sucking insects inflict less mechanical damage than leaf chewing or mining and boring insects. However, some sap-sucking insect species may transmit viruses or cause deformation and stunting of shoots (Schoonhoven et al., 2005).

Gall-induction is another feeding habit of herbivorous insects. Generally, galls are defined as pathologically developed cells, tissues, or organs of plants that have arisen by hypertrophy (increase in cell size) and/or hyperplasia (increase in cell number) as a result of stimulation from foreign organisms (Redfern, 1997; Raman, 2012). The formation of the gall is believed to be beneficial to the insects, rather than a defensive response of the plant to insect attack (Stone and Schönrogge, 2003). Most galls serve as sinks of plant assimilates, thereby, providing high quality food to the insect (Bagatto et al., 1996; Koyama et al., 2004). Galls also provide a protective microenvironment to sedentary feeders such as aphids and psyllids compared to normal plant surfaces. Some galls are also known for protecting certain insects from their parasitoids (Gullan and Cranston, 2010). Usually galls are initiated from young leaves, flower buds, stems, and roots. They are rarely initiated on mature plant parts. Continued stimulation of the cells of the plants by the insect determines the development and growth of insect-induced galls. The involvements of oral secretions, anal excreta, and accessory gland secretions have been emphasized in the initiation and growth of galls. Salivary substances such as amino acids, growth regulators, phenolic compounds, and phenol oxidases may have a role in the formation of galls or in overcoming the plant defense (Schoonhoven et al., 2005; Gullan and Cranston, 2010). The involvement of plant hormones such as auxins and cytokinins in the formation of galls is very likely, though it is not clear yet whether such hormones are produced by the insect or the plant under attack (Raman, 2012). Genetic entities such as viruses, plasmids, or transposons, which can be transferred from the insect to the plant, may also play role in the formation of certain complex galls (Gullan and Cranston, 2010).

## MOLECULAR MECHANISMS OF PLANT IMMUNITY

Plants have several preformed physical (e.g., wax layer, trichomes) and chemical (toxins) barriers, to ward off pathogens and herbivores. In case these barriers are overcome by the attackers, plants activate a multilayered innate immune system to suppress the infection (Jones and Dangl, 2006). Most of our knowledge about the plant immune system is derived from studies on plant interactions with pathogens (bacteria, viruses, fungi). Now that insights in the herbivore–plant and nematode–plant interactions begin to appear, it becomes clear that although there are several specificities in these interactions, also considerable similarities with pathogen-induced plant responses exist (Kaloshian and Walling, 2005). Therefore, the knowledge about the molecular mechanisms involved in plant responses to pathogens cannot only help to interpret the observations on nematode and herbivore plant interactions, but also provide inspiration for new experiments. Hence, before discussing the nematode–plant and herbivore–plant interactions, we give a brief overview of the basic mechanisms of plant immunity against pathogens.

The first layer of plant’s innate immunity consists of a system that is directed against so called pathogen- or microbe-associated molecular patterns (PAMPs or MAMPS), which are conserved molecules characteristic for a big phylogenetic group of pathogenic and non-pathogenic microbes and are often located at the external surface (e.g., fungal chitin or bacterial lipopolysaccharides). PAMP-triggered immunity (PTI) starts with detection of these PAMPs by pattern recognition receptors, which are transmembrane proteins located at the cell surface. They typically consist of an extracellular leucine-rich-repeat, a transmembrane domain, and a cytoplasmic kinase domain (Nurnberger and Kemmerling, 2006; Zipfel, 2008; Monaghan and Zipfel, 2012). Binding of these immune receptors to PAMPs causes a cellular signal cascade of ion influxes and mitogen-activated protein (MAP) kinase activation resulting in production of reactive oxygen species, changes in gene expression and cell wall reinforcements (Schwessinger and Zipfel, 2008; Antolin-Llovera et al., 2012; Schwessinger and Ronald, 2012). In response to the plant defenses, pathogens and herbivores have in turn evolved several mechanisms to evade or suppress the plant’s innate immune system (Jones and Dangl, 2006). This is accomplished by secretion of proteins, the so-called “effectors.” Besides evasion or suppression of the host defense system, effectors can also be involved in manipulation of the host developmental program, for example when galls are formed (Gheysen and Mitchum, 2011). In the course of evolution, plants have developed a second layer of immunity that responds to the presence of these effectors and is called Effector-triggered immunity (ETI). The immune receptors for ETI, called resistance (R) proteins, consist of a leucine-rich-repeat domain attached to a nucleotide binding domain with a coiled–coiled or toll-interleukin receptor N-terminal domain (van Ooijen et al., 2007). They are mostly located inside the cytoplasm, but a few also reside on the plasma membrane with their leucine-rich-repeat facing the apoplast. ETI results in a very fast defense response at the site of invasion, which is marked by a rapid calcium and potassium influx, activation of MAP kinase pathways, formation of reactive oxygen species, and ultimately a local programmed cell death, also known as the hypersensitive response (Jones and Dangl, 2006; Spoel and Dong, 2012). Neighboring cells respond by producing toxic compounds and strengthening of their cell walls. An ETI response is much stronger than a PTI response and often blocks the pathogen at the site of invasion.

Originally, it was thought that R proteins interact directly with a certain effector. Because effectors are very species specific, ETI would only be effective against closely related strains of pathogens or herbivores, while PTI is directed against a broader phylogenetic range of pathogens and herbivores possessing conserved PAMPs. Although there are some examples of R proteins directly interacting with effectors, recent insights suggest that the majority of the R proteins monitor modifications caused by the effectors on own proteins (Jones and Takemoto, 2004; van der Hoorn and Kamoun, 2008). This guarding of self-proteins has the advantage that with a limited number of R proteins the activity of numerous effectors can be sensed. Indeed, whole genome sequencing of several plant species demonstrated that the number of different R proteins is much smaller than the number of effectors that they can encounter after attack by different species of pathogens and herbivores (Goff et al., 2002; Meyers et al., 2003). Protein interaction studies have also shown that effectors tend to be mostly directed against the same “hubs” in the immune reaction signaling pathway (Mukhtar et al., 2011), and therefore guarding the modification of a limited number of these hub self-proteins by the R proteins is sufficient to provide resistance against a broad spectrum of invaders.

Induction of PTI and ETI also lead to activation of hormonal signaling pathways, such as the salicylic acid (SA), jasmonic acid (JA), and ethylene (ET) pathway. In general, the SA pathway is induced by biotrophic pathogens, while the JA and ET pathways are induced by wounding or necrotrophic pathogens (Pieterse and van Loon, 1999). However, recent experiments with the pathogen *Pseudomonas syringae* have demonstrated that all three hormonal pathways are important for PTI as well as ETI (Tsuda and Katagiri, 2010). The authors propose a model whereby plants would initially activate all three pathways at low levels, and when the pathogen remains, priority would be given to the most effective pathway only later (Katagiri and Tsuda, 2010). Crucial in this model are indications that the three hormonal pathways consist of several sectorial signaling cascades, whereby some of these sectors are shared between the different hormonal pathways. Depending on the kind of pathogen, the plant would strongly activate those signaling sectors that are the most efficient in suppressing the intruder. It’s an intriguing idea and would explain why each pathogen or herbivore species seems to induce a different but partially overlapping transcriptome profiles that strongly depend on their feeding habit (De Vos et al., 2005; Bidart-Bouzat and Kliebenstein, 2011). Although it still needs to be investigated whether this holds true for other pathogens and herbivores as well, this initial low level activation of all three pathways could have significant implications for consecutive infections with other pathogen species.

## SYSTEMIC INDUCED RESISTANCE

In addition to the local PTI and ETI responses, a systemic response occurs. This systemic response, for which the signal is not always known, can either directly alter the defensive state of the undamaged organs, or it can “prime” or prepare distant tissues for upcoming attacks. Typical for the primed state is a stronger and faster cellular immune response after a second infection (Conrath et al., 2002, 2006). While the exact molecular mechanism of priming is not completely resolved yet, it probably is based on epigenetic modifications that suppress or enhance the transcription of key regulators of the immune response (Bruce et al., 2007; van den Burg and Takken, 2009). A well-known example of priming is the enhanced broad spectrum resistance against pathogens and herbivores after infection with beneficial soil microorganisms, such as plant growth promoting bacteria and mycorrhizal fungi (Van Wees et al., 2008).

Similar to the local responses, systemic induced defense responses are mainly controlled by the plant hormones SA, JA, and ET (Pieterse et al., 2012). Recently, it was discovered that the JA pathway consists of two antagonistic branches (Verhage et al., 2011). The first branch, activated by herbivorous insects, is controlled by the MYC2 transcription factor and – in *Arabidopsis thaliana *– is characterized by the strong induction of the marker gene *VSP2*. The second branch, called the ERF branch, provides resistance against necrotrophic pathogens in *A. thaliana* and is controlled by the ORA59 transcription factor with *PDF1.2* as the marker gene.

Considerable cross-talk occurs between the different hormonal pathways (Bostock, 2005; Pieterse et al., 2012). In general, SA is known to suppress the JA pathway (Spoel et al., 2003). This can lead to trade-offs in the defense responses when plants are attacked simultaneously by different pathogens. For instance, prior infection with the SA-inducing biotrophic pathogen *Hyaloperonospora arabidopsidis *suppresses the JA-controlled defense against caterpillars of *Pieris rapae* (Koornneef et al., 2008). However, this antagonistic interaction between SA and JA seems to be dependent on the concentration of both hormones, whereby a low concentration of both results in a synergistic effect, while high concentrations lead to antagonism (Mur et al., 2006). The SA–JA antagonism seems also to be dependent on the time that has passed between the induction of both hormonal pathways (Koornneef et al., 2008). Moreover, ET signaling prevents SA-mediated suppression of the JA pathway (Leon-Reyes et al., 2010).

Systemic induced defense signaling ultimately results in the activation of a wide range of different defensive traits. These could be morphological changes (e.g., formation of trichomes), production of defensive proteins (e.g., chitinase, proteinase inhibitors) or toxins (phytoalexins, alkaloids, glucosinolates), or release of volatiles that either have a repellent effect or attract predators of the attacking herbivores (Bezemer and van Dam, 2005; Kaplan et al., 2008a; Dicke et al., 2009).

## PLANT DEFENSE AGAINST NEMATODES

### LOCAL DEFENSES AGAINST NEMATODES

Because many PPN species invade the host and remain inside for several weeks to months, they inevitably expose themselves to being detected by PAMP immune receptors. The nematode body is protected on the outside by a cuticle consisting of highly conserved collagens, which may serve as cues (epitopes) for the plant’s defense system. However, until now no PAMP receptors have been identified that are directed against conserved epitopes of PPN. Most likely, this is due to several strategies developed by nematodes to evade or suppress PTI (Nobre and Evans, 1998; Davies and Curtis, 2011). First, once inside the plant root, the developing nematodes undergo three consecutive molts. The composition of the new cuticle changes after each molt, thereby creating a new challenge to the plant immune system. Second, the nematode cuticle is covered by a carbohydrate-rich surface coat that is constantly shed and changes in composition, thereby creating a moving and variable target for the plant immune system. Third, the surface coat contains lectin-like proteins, which are capable of binding plant carbohydrates (Spiegel et al., 1995). Although not directly demonstrated, it might well be that, just like animal parasitic nematodes (Blaxter et al., 1992; Maizels et al., 2001), PPN cover themselves with host derived carbohydrates and thereby prevent being recognized by the plant immune system as a non-self-entity.

Despite the strategies to avoid recognition, nematode invasion also activates the plant’s immune response. As early as 12 h after root penetration by the root-knot nematode *Meloidogyne incognita*, which is still during the root migration phase (Wyss et al., 1992), peroxidases, cell wall modification enzymes, *LOX *genes, and proteinase inhibitors were induced (Gheysen and Fenoll, 2002). Production of reactive oxygen species, callose deposition, and cell wall thickening were also observed during cyst nematode root migration (Waetzig et al., 1999). Whether these resemble typical wounding responses controlled by JA or PTI induced by PAMP detection remains to be investigated. Because endoparasitic PPN are armed with a robust stylet and a cocktail of cell wall degrading enzymes, cell wall thickening is certainly not sufficient to provide complete resistance against PPN. Nevertheless, root inoculation experiments with sedentary endoparasitic juveniles clearly show that only a fraction of them succeeds in penetrating the roots and reach the vascular cylinder where they can induce a feeding site (Wyss et al., 1992; Tytgat et al., 2002). Moreover, even a weak PTI defense might generate a first systemic signal and prime for a stronger defense at later time points against the same or different pathogens or herbivores.

Numerous effectors have been identified in different PPN by RNA profiling, EST (expressed sequence tag) or whole genome sequencing (Vanholme et al., 2004; Abad et al., 2008; Davis et al., 2008; Opperman et al., 2008; Bellafiore and Briggs, 2010; Haegeman et al., 2012). In a recent experiment, mass spectrometric analysis of nematode secretory proteins identified 486 proteins secreted by pre-parasitic *M. incognita *juveniles, which illustrates the complexity of the effector repertoire used by nematodes (Bellafiore et al., 2008). Numerous effector proteins are cell wall degrading enzymes such as cellulases, pectate lyases, polygalacturonases, xylanases, and expansins. They are found in ectoparasitic, migratory, and sedentary endoparasitic nematodes and are involved in cell wall softening mainly during root migration. Several secreted enzymes, such as glutathione peroxidase and peroxiredoxin, protect against reactive oxygen species that may be formed in response to infestation (Jones et al., 2004; Dubreuil et al., 2011). In the potato cyst nematode *Globodera rostochiensis* a protein called SPRYSEC19 was identified that blocks the activation and consecutive hypersensitive response of several know resistance proteins (Postma et al., 2012). Other effectors are thought to suppress SA or JA production or interfere with the plant’s ubiquitin-proteasome pathway (Haegeman et al., 2012). The latter is a mechanism that is often used by pathogens to suppress the plant immune system (Angot et al., 2007). Specific for the sedentary nematodes are also numerous proteins that are thought to be involved in feeding cell induction (Davis et al., 2008; Gheysen and Mitchum, 2011; Haegeman et al., 2012).

Several R genes are identified in different plant species that render resistance against sedentary endoparasitic cyst and root-knot nematodes (Tomczak et al., 2009). None of them seems to be effective during the root migration phase of the nematode, but rather block the development of the feeding site, where after the nematode dies due to starvation (Bakker et al., 2006). Interestingly, the tomato Mi-1 R protein that renders resistance to root-knot nematodes (*Meloidogyne *spp.) also provides resistance to potato aphids (*Macrosiphum euphorbiae*) and whiteflies (*Bemisia tabaci*; Rossi et al., 1998; Vos et al., 1998; Nombela et al., 2003). It was demonstrated that this ETI resistance requires SA, but not JA (Branch et al., 2004; Li et al., 2006; Bhattarai et al., 2007, 2008). Strikingly, host plants without the *Mi-1* gene were more susceptible in a choice experiment to potato aphids when the JA pathway was blocked (Bhattarai et al., 2007). However, no effect of JA on the fecundity or survival of the potato aphids was found. This illustrates that in the absence of *Mi-1-*conferred ETI, plants still can have another form of defense, in this case a JA-dependent defense that renders them less attractive to the aphids.

### SYSTEMIC INDUCED RESPONSES AFTER NEMATODE INFESTATION

While several gene expression studies have been performed on PPN-infected plants, most of them were designed to analyse the local response, mainly feeding site development of sedentary endoparasitic nematodes. Only recently a few studies have been published providing information on the systemic induced defense after PPN infection. A microarray analysis of *A. thaliana *after infection with the cyst nematode *Heterodera schachtii* revealed a strong induction of *VSP2*, a marker gene for the MYC2 branch of the JA defense pathway, in the whole root system at day 3 after nematode inoculation (Puthoff et al., 2003). At that time point, juveniles have penetrated the roots and reached the vascular cylinder where they just have started inducing the feeding site. Transcriptome analysis during a time course experiment (6 h till 8 days after infection) of soybean with the soybean cyst nematode, *Heterodera glycines*, also found a clear induction of the JA pathway in the whole root system at all time points (Alkharouf et al., 2006). A similarly clear systemic induction of the JA pathway in soybean roots was observed at day 2, 5, and 10 after *H. glycines *infection (Ithal et al., 2007a), however, locally in the developing syncytia the JA-controlled defense was suppressed (Ithal et al., 2007b).

A comparison of systemic defense signaling after rice infection with a root-knot nematode and a migratory endoparasitic nematode was performed (Kyndt et al., 2012). Infection with the migratory endoparasitic nematode *Hirschmanniella oryzae* activates a systemic JA and ET signaling at day 3, while the SA pathway is suppressed. However, by day 7 the JA and ET signaling is repressed again. At day 3, infection with the root-knot nematode *Meloidogyne graminicola *activates in the systemic root tissue SA and JA, but suppresses ET. By day 7, also the JA pathway is largely suppressed. In contrast, in the shoot tissue all three hormonal defense pathways are suppressed already at day 3 by this nematode. Foliar application of the hormones SA, JA, or ET and mutants analysis showed that *M. graminicola *is mainly sensitive to a JA- and ET-induced defense, but only slightly to SA-induced defense, while *H. oryzae* was sensitive to all the defenses controlled by all three hormones (Nahar et al., 2011; Nahar et al., 2012). In tomato plants, spraying with methyl jasmonate also results in a lower infection rate of *M. incognita* (Fujimoto et al., 2011).

Similar observations of an early shoot defense suppression after *M. incognita *infection were performed in *A. thaliana* where several marker genes for the SA and JA pathway were measured after 5 till 14 days (Hamamouch et al., 2011). In the roots, *M. incognita *infection of *A. thaliana *strongly induces the SA-controlled defense at day 9 and 14, but not at day 5. In addition, a weak response of JA-controlled defense markers was observed at day 9. *A. thaliana *infection with the cyst nematode *H. schachtii* strongly induces the SA, but not the JA marker genes in the roots starting from day 5. In the shoots, however, also some JA marker genes are induced. Moreover, SA-deficient *A. thaliana *mutants exhibit an increased susceptibility to *H. schachtii, *whereas ectopic application of SA renders wild type plants less susceptible (Wubben et al., 2008). In conclusion, sedentary endoparasitic nematodes seem to initially induce the JA, ET, and SA pathways, but very quickly, especially after *M. incognita *infection, parts of these pathways are repressed again.

## PLANT DEFENSES AGAINST INSECTS

Insect herbivore-induced immune responses have been reviewed extensively recently (Hilker and Meiners, 2010; Wu and Baldwin, 2010; Arimura et al., 2011; Bonaventure et al., 2011; Hogenhout and Bos, 2011; Kim et al., 2011; Erb et al., 2012; Kerchev et al., 2012; Mithofer and Boland, 2012; Smith and Clement, 2012). Therefore, we only summarize the main points that are essential for understanding how interactions between root-feeding nematodes and shoot-feeding herbivores may occur.

Similar to pathogens, insect herbivores are also detected in two ways. First, wounding by chewing insect causes a release of cellular components that are otherwise compartmentalized. Some of these components act as elicitors of defense reactions and are called damage-associated molecular patterns (DAMPS; Boller and Felix, 2009; Koo and Howe, 2009). Second, herbivore elicitors present in oral or ovipositor secretions, called herbivore-associated molecular patterns (HAMPS), are detected by the plant (Mithöfer and Boland, 2008).

Both DAMPS and HAMPS may trigger the production of a plethora of plant defense responses, ranging from increases in morphological defenses, such as trichomes (Mathur et al., 2012) or chemical defense, such as phenolics, alkaloids, terpenoids, or glucosinolates (van Dam et al., 2009; Turlings et al., 2012). These induced responses may directly contribute to plant resistance by deterring herbivore feeding or indirectly by attracting the herbivores’ enemies, e.g., predators and parasitoids, to the plant (Dicke et al., 2009). Which of the many defense compounds are produced upon damage – and thus the effect on the herbivore or its parasitoids – is determined early in the induction process by cross-talk between the JA, SA, and ET pathways. It is thought that cross-talk between signaling pathways is essential for the fine-tuning of plant responses to specific attackers. Each herbivore species may elicit a specific signal signature, which triggers the transcription of distinctive sets of genes (De Vos et al., 2005; Diezel et al., 2009; Verhage et al., 2011). The feeding strategy of the herbivores may greatly co-determine the transcription profile after induction: both specialist and generalist aphids induce transcription profiles in *A. thaliana* that are more similar to each other than to the transcription profiles induced by different species of caterpillars (Bidart-Bouzat and Kliebenstein, 2011). Similar as for pathogen-induced defense responses, herbivore-induced defense responses are often systemic thus affecting the preference and performance of other herbivores feeding elsewhere on the plant (Bezemer and van Dam, 2005; Kaplan et al., 2008a, b; Rasmann et al., 2011).

## CHANGES IN PRIMARY METABOLISM

In addition to the increased production of defense compounds, herbivore- and pathogen-induced responses may also alter the plant’s primary metabolism, and, consequently, the preference and performance of root and shoot herbivores feeding on the plant. Several recent studies investigated the induced changes of resource allocation after herbivore attack or application of defense-related hormones. Radio-actively labeled CO_2_ was used to track assimilated carbon in *Populus *spp. after JA treatment of the leaves (Babst et al., 2005). An increased export of photosynthate toward the stem and roots was observed. An increased allocation of photosynthate to the roots was also observed in *Nicotiana attenuata *after simulated herbivory (Schwachtje et al., 2006). In tomato, JA treatment of the leaves resulted in an increased export of photosynthate and amino acids out of the treated leaves and resulted in an increased amino acid content in the roots (Gómez et al., 2010).

A comparison of leaf and root JA application showed that leaves had a lower total sugar and amino acid concentration after leaf JA application, but only a lower total amino acid concentration after root JA application (van Dam and Oomen, 2008). Measurement of 56 primary metabolites in different tissues of tomato after herbivory with two caterpillar species demonstrated rapid changes that are tissue and herbivore species specific (Steinbrenner et al., 2011). Also PPN seem to influence the primary metabolism of its host. Metabolic analysis after infection with the cyst nematode *H. schachtii* resulted in decreased amino acid levels in the shoots, but a higher concentration of glyceric acid, gluconic acid, trehalose, 1-kestose, and raffinose (Hofmann et al., 2010).

It is suggested that this change in primary metabolites after herbivore or pathogen infection can have several reasons (Schwachtje and Baldwin, 2008): (i) primary metabolites are used to synthesize defensive secondary metabolites (Smith and Stitt, 2007; Bolton, 2009), (ii) reallocation of resources away from the site of attack my safeguard them for future plant regrowth (Utsumi and Ohgushi, 2007; Steinbrenner et al., 2011), (iii) primary metabolites, such as trehalose, may serve as a signal in the defense pathway (Ahn and Lee, 2003; Bolton, 2009), (iv) primary metabolites may have a defensive function themselves (Lou and Baldwin, 2004). Moreover, recent findings indicate that insect herbivory reduces photosynthesis by transcriptional reprograming as well as physiological mechanisms (Kerchev et al., 2012). We therefore suggest that when investigating plant-mediated interactions between different pathogens or herbivores, not only defense mechanisms should be considered, but also changes in primary metabolism. For instance, a change in the amino acid or sugar content of the phloem sap could have significant effects on aphid performance.

## ABOVEGROUND–BELOWGROUND INTERACTIONS BETWEEN NEMATODES AND INSECT HERBIVORES

As discussed above, there is clearly a scope for interactions between belowground PPN and aboveground herbivores feeding on the same plant. Most of the plant responses induced by either of these herbivore classes are systemic and moreover the induced responses are governed by the same signaling pathways. The feeding strategy of the nematodes, the degree of host plant susceptibility to herbivores and the identity of shoot herbivorous insects have been suggested as important factors determining the outcome of interactions between root-feeding nematodes and shoot herbivorous insects as these may differentially influence the responses of the host plants to attackers (Mateille, 1994; van Dam et al., 2003, 2005; Bezemer and van Dam, 2005; Wurst and van der Putten, 2007). The penetration, migration, induction of feeding sites, formation of lesions, cell death, and other infection processes caused by nematodes and the different feeding habits of herbivorous insects (leaf chewing, sap-sucking, leaf mining and boring, and gall-induction) may elicit different hormonal regulatory responses in their host plants. These differential host responses in turn may lead to different systemic induced responses and thereby have differential effects on herbivores feeding on the other plant compartment. Based on what is currently known about plant responses to different species of nematodes, it has been postulated that migratory endoparasitic and ectoparasitic nematodes influence the host plant’s immune system to a lesser extent than sedentary endoparasites (Mateille, 1994; Zinov’eva et al., 2004; Bezemer and van Dam, 2005). This would mean that the effects of sedentary nematodes on aboveground herbivores would be greater than that of migratory nematodes. However, there is little concrete experimental evidence that this is the case. Here, we rather suggest that each nematode species can significantly affect their host plant’s defense equally strong, but that the direction of the effects on the aboveground herbivores differ depending on the feeding strategy and species of the nematode.

In the last decade, empirical evidence on the occurrence of plant-mediated interactions between root-feeding nematodes and aboveground herbivorous insects are accumulating (**Table 1**). van Dam et al. (2005) reported that the quality of black mustard, *Brassica* nigra, for the shoot herbivore *Pieris rapae* decreased as a result of root herbivory by the migratory endoparasitic nematode *Pratylenchus penetrans. *The reduced performance of the caterpillar was attributed to the enhanced production of phenolics and glucosinolates following root- and shoot-feeding (van Dam et al., 2005).

**Table 1:** Overview of the plant-mediated interaction studies between root-feeding nematodes and leaf herbivorous insects, whereby a distinction between the different feeding habits is made.

Plant	Nematode		Insect		Outcome	Suggested mechanism	Reference
	Migratory	Sedentary	Leaf chewer	Phloem feeder
*Brassica nigra*	*Pratylenchus penetrans*	–	*Pieris rapae*	–	Reduced caterpillar performance	Enhanced production of phenolics and glucosinolates	van Dam etal. (2005)
*Nicotiana tabacum*	*Tylenchorhynchus *sp., *Xiphinema* sp. (ectoparasites) *Pratylenchus* sp.	–	*Spodoptera exigua*	–	Higher number of nematodes on insect defoliated plants	–	Kaplan etal. (2009)
*Plantago lanceolata*	*Pratylenchus penetrans*	–	–	*Myzus persicae*	Reduced aphid fecundity	Systemic induced defense, change in nutritional quality	Wurst and van der Putten (2007)
*Nicotiana tabacum*	*Tylenchorhynchus *sp., *Xiphinema* sp. (ectoparasites) *Pratylenchus* sp.	–	–	*Myzus persicae*	Negative correlation between nematodes and aphids	–	Kaplan etal. (2009)
*Anthoxanthum odoratum*	Paratylenchidae and Dolichodoridae (ectoparasites) Pratylenchidae	–	–	*Rhopalosiphum padi*	Reduced aphid fecundity	Reduced amino acid concentrations in phloem sap	Bezemer etal. (2005)
*Agrostis capillaris*	Paratylenchidae and Dolichodoridae (ectoparasites) Pratylenchidae	–	–	*Rhopalosiphum padi*	Reduced aphid fecundity	Reduced amino acid concentrations in phloem sap	Bezemer etal. (2005)
*Phaseolus vulgaris*	*Pratylenchus penetrans*	–	–	*Tetranychus urticae* (cell content feeder)	Reduced spider mite fecundity	–	Bonte etal. (2010)
*Ammophila arenaria*	*Pratylenchus brzeskii*	*Meloidogyne *sp. *Heterodera *sp.	–	*Schizaphis rufula*	Microcosm: lower aphid preference for nematode-infected plants Field: no effect	In fields: additional parameters influence herbivores	Vandegehuchte etal. (2010)
*Glycine max*	–	*Heterodera glycines*	–	*Aphis glycines*	Lower aphid preference for nematode-infected plants	Change in volatile emissions	Hong et al. (2010, 2011)
*Brassica oleracea*	–	*Heterodera schachtii*	–	*Brevicoryne brassicae*	Reduced aphid growth and fecundity	–	Hol etal. (2010)
*Beta vulgaris*	–	*Heterodera schachtii*	–	*Myzus persicae*	Reduced aphid growth and fecundity	–	Hol etal. (2010)
*Brassica oleracea*	mix of nematode species		–	*Brevicoryne brassicae*	No effect on aphid population growth	–	Kabouw etal. (2011)
*Arabidopsis thaliana*	–	*Heterodera schachtii*	–	*Brevicoryne brassicae*	No effect on aphid performance Reduced nematode infection	Aphid induced changes in glucosinolate content	Kutyniok and Muller (2012)
*Nicotiana tabacum*	–	*Meloidogyne incognita*	–	*Myzus persicae*	Lower aphid growth rate and fecundity No effect on nematode performance	Systemic induced defense	Kaplan etal. (2011)
*Glycine max*	–	*Heterodera glycines*	*Helicoverpa zea*	–	Increased number of insect larvae on nematode-infected plants	–	Alston etal. (1991)
*Glycine max*		*Meloidogyne incognita*	*Pseudoplusia includens*	–	No effect on caterpillar development	–	Carter-Wientjes etal. (2004)
*Nicotiana tabacum*	–	*Meloidogyne incognita*	*Trichoplusia ni* (generalist)	–	Increased caterpillar performance	Interference with nicotine synthesis and transport to the shoots	Kaplan etal. (2008a)
*Nicotiana tabacum*	–	*Meloidogyne incognita*	*Spodoptera exigua* (generalist)	–	Increased caterpillar performance	Interference with nicotine synthesis and transport to the shoots	Kaplan etal. (2008a)
*Nicotiana tabacum*	–	*Meloidogyne incognita*	*Manduca sexta* (specialist)	–	No effect on caterpillar	Specialist insect not sensitive to nicotine	Kaplan etal. (2008a)
*Gossypium hirsutum*	–	*Meloidogyne incognita*	*Helicoverpa zea*	–	No effect on caterpillar	No induction of toxins, no increased attraction of parasitoids	Olson etal. (2008)
*Glycine max *	–	*Heterodera glycines*	*Pseudoplusia includens*	–	Increased nematode performance	–	Russin etal. (1989)
*Glycine max*	–	*Meloidogyne incognita*	*Pseudoplusia includens*	–	Increased nematode performance	–	Russin etal. (1993)
*Glycine max*	–	*Heterodera glycines*	*Helicoverpa zea*	–	Increased nematode performance	Longer growth season of host plant due to defoliation	Alston etal. (1993)
*Zea mays*	Mix of nematode species		*Romalea guttata*	–	No effect on nematodes	–	Fu etal. (2001)
*Zea mays*	–	*Meloidogyne incognita*	*Ostrinia nubilalis* (stalk borer)	–	Reduced nematode root penetration	Systemic induced defense	Tiwari etal. (2009)

In a microcosm experiment, the PPNs, which mainly consisted of ectoparasites and migratory endoparasites, caused a reduced fecundity of the aphids *Rhopalosiphum padi* feeding on *Agrostis capillaris* and *Anthoxanthum odoratum *(Bezemer et al., 2005). A lower amino acid content was observed in the phloem sap of the nematode-infected plants, and was probably one of the causes of this decreased aphid performance. A study on *Plantago lanceolata *also documented negative aphid–nematode interactions with lower aphid fecundity on plants attacked by the nematode *P. penetrans *(Wurst and van der Putten, 2007)*.* Herbivory by *P. penetrans* decreased the number of offspring produced by the aphid *Myzus persicae *by 43.8%. The authors suggested that the nematodes might have affected the aphids via changes in the nutritional quality of the aboveground plant parts and possibly via the induction of the same plant defense pathways as those induced by aphids. The latter idea finds support in the fact that the *Mi-1* gene confers resistance to root-knot nematodes as well as aboveground sucking insects such as white flies and aphids (Vos et al., 1998; Kaloshian, 2004). The possibility of priming is also raised as the earlier infection with nematodes might have enhanced the defense against the subsequent attackers (aphids). Also cell content feeding spider mites (Arthropoda) showed a reduced fecundity when feeding on *P. penetrans*-infected *Phaseolus vulgaris *plants (Bonte et al., 2010).

The influence of an infection with sedentary endoparasitic nematodes on sap-sucking insects is rather variable. An infection with *H. schachtii *of *Beta vulgaris *or *Brassica oleracea *resulted in a reduced growth and fecundity of *Brevicoryne brassicae *and *M. persicae *(Hol et al., 2010)*. M. persicae *also showed a reduced growth rate and fecundity when feeding on *M. incognita*-infected *Nicotiana tabacum *(Kaplan et al., 2011). In contrast, no effect on the performance of *B. brassicae *was found by an infection of *B. oleracea *with a mix of different root parasitic nematode species (Kabouw et al., 2011). In *A. thaliana*, a simultaneous inoculation with the sedentary endoparasitic nematode *H. schachtii* and the aphid *B. brassicae* was performed (Kutyniok and Muller, 2012). No effects of the nematode infection were observed on the aphid performance at day 3. In contrast, a lower number of nematodes were found on the aphid-infested plants compared to control plants. Hong et al. (2010) studied the effects of soybean plant infection with soybean cyst nematode (*H. glycines)* on the preference and performance of soybean aphid (*Aphis glycines*; Hong et al., 2011). Soybean aphids prefer uninfected plants compared to plants infected with soybean cyst nematode. However, the effect of soybean cyst nematode on the performance of aphids was not significant in one set of experiments and significant in another experiment. Ultimately, the authors concluded that soybean cyst nematode primarily influences the behavior of soybean aphid more than its performance. Similarly, in a controlled microcosm experiment conducted on Marram grass, *Ammophila arenaria*, the aphid *Schizaphis rufula *showed a lower preference for plants infected with mix of *Pratylenchus brzeskii*, *Meloidogyne *and *Heterodera *sp. (Vandegehuchte et al., 2010). However, in the field no significant correlations between the abundances of the two groups of herbivores were detected. The authors argue that in the field, other variables related to plant vitality and water content structure herbivore populations.

Different results were also obtained when studying the plant-mediated effects of sedentary endoparasitic nematodes on leaf chewing insects. In a field study, a higher number of *Helicoverpa zea *larvae was found when *Glycine max *plants were infected with *H. glycines* (Alston et al., 1991). Kaplan et al. (2008a) reported that belowground herbivory by the root-knot nematode *M. incognita *on tobacco plants increased the larval weight of the aboveground generalist caterpillar *Trichoplusia ni* by 29%, whereas herbivory by the same nematode did not significantly affect the performance of the specialist caterpillar *Manduca sexta*. As plants in the genus *Nicotiana* produce alkaloids such as nicotine for constitutive and induced defense against aboveground herbivores (Steppuhn et al., 2004), this facilitation effect of nematode herbivory on aboveground herbivorous insects may result from interference of nematode feeding with the transport and biosynthesis of nicotine which takes place in the roots. This is supported by the higher (>2 times) concentration of leaf nicotine in control plants when compared to *M. incognita*-infested plants (Kaplan et al., 2008a). Furthermore, nematode root herbivory on nicotine producing plants increases the weight gain of the caterpillar *Spodoptera exigua*, whereas the performance of the caterpillar on nicotine-deficient plants is not affected as a result of root herbivory by nematodes (Kaplan et al., 2008a). The same authors have also noticed that nematode-free plants respond to caterpillar feeding by inducing higher levels of nicotine whereas nematode-infected tobacco plants are impaired in their ability to induce nicotine levels upon larval feeding.

Contrary to the above cases, Olson et al. (2008) reported that root herbivory by the root-knot nematode *M. incognita* has little influence on the direct and indirect induced defense of cotton, *Gossypium hirsutum*, against insect herbivory. In this study, the levels of gossypol and gossypol-like compounds, and the emissions of induced local and systemic volatiles were measured in cotton plants that are exposed to either the foliar feeder *H. zea*, the root-feeding nematode *M. incognita *or their combination. The attraction of the parasitic wasp *Microplitis croceipes* to plants exposed to the different treatments was also investigated. Local and systemic induction of volatiles that attract the parasitoid *M. croceipes *occurred two days after leaf herbivory, and an increased level of herbivore-induced volatiles was recorded from plants infested by nematodes. However, these differences in induced volatile emissions did not affect the attraction of *M. croceipes*: plants with nematodes and an aboveground herbivore were equally attractive as plants with caterpillar damage only. None of the treatments led to changes in gossypol and gossypol-like compounds in leaf or root tissues. Also no effects were found on *Pseudoplusia includens* caterpillar development by a *M. incognita *infection on *Glycine max *(Carter-Wientjes et al., 2004).

While the above examples illustrate the influence of PPN on aboveground feeding insects, leaf feeding insects in turn can also influence PPN performance. Using field surveys and experimental field studies, it was demonstrated that nematode performance was influenced by the aboveground insect feeding guild on tobacco plants (Kaplan et al., 2009). This study shows that positive interactions between nematodes and leaf chewing insects (e.g., caterpillars) predominate, whereas negative interactions occur with sap-feeding insects (e.g., aphids). Overall, insect defoliated plants had a 41% higher numbers of tobacco feeding nematodes in the rhizosphere compared to insect-free plants (Kaplan et al., 2009). The total numbers of nematodes were lower in the rhizosphere of aphid-infested plants, but the effects differed between nematodes species. The ectoparasitic nematode *Tylenchorhynchus* was less abundant whereas the density of *Pratylenchus *remained unaffected by aphid herbivory. Similarly, an increased number of *H. glycines *and *M. incognita* were found on *G. max *when they were defoliated by *P. includes* or *H. zea* caterpillars (Russin et al., 1989; Alston et al., 1993; Russin et al., 1993). In contrast, stalk boring by *Ostrinia nubilalis *resulted in a reduced number of *M. incognita *penetrating the roots of *Zea mays *(Tiwari et al., 2009), while defoliation by *Romalea guttata* had no effect on the number of nematodes (Fu et al., 2001).

The above mentioned plant-mediated interactions between root-feeding nematodes and leaf herbivorous insects are summarized in **Table 1**. Although many more data are needed to draw any real conclusions, the outcome of the interaction seems indeed to be at least partially determined by the feeding habits. For instance, migrating endoparasitic nematodes (e.g., *Pratylenchus *sp.) cause a reduced aphid fecundity, while sedentary endoparasitic nematodes (e.g., *Heterodera, Meloidogyne*) rather decrease the attractiveness of the plants for these sap-sucking insects, but can have a variable influence on the aphid performance. This example illustrates also that when studying plant-mediated interactions between nematodes and insects all different possible plant responses against infestation should be considered. It would for instance be interesting to know how a nematode infection influences the oviposition preference of Lepidoptera. Moreover, because indirect defenses, such as attraction of parasitoids and predators, are important mechanisms of plants to deal with herbivore infections, plant-mediated interactions between nematodes and herbivorous insects should preferably be studied in a multitrophic environment.

## CONCLUSIONS AND FUTURE OUTLOOK

Given the high abundance of both groups of herbivores in the field, plants will inevitably encounter both root-feeding nematodes and aboveground feeding insects in their lifetime. Both types of herbivores will elicit induced defenses responses, and possibly also shifts in primary metabolites, that are systemic throughout the plant. It is therefore likely that PPN and aboveground insects interact with each other via systemic induced responses in the plant. The evidence for these interactions is slowly accumulating. Given the observed induction of SA, JA, and ET defense signaling pathways and a partial repression of them later on by the sedentary PPN, it is hard to predict the outcome of a combined infection with PPN and herbivorous insects. Therefore, a more detailed analysis of the plant-mediated interactions between PPN and insects is necessary to better understand how plants integrate the induced responses that are triggered by these different groups of herbivores. This knowledge will also lead to new insights in the regulation of plant-induced responses under multiple attacks, as is common in natural environments. Moreover, analyzing the molecular responses of plants challenged with a combined infection of root parasitic nematodes and shoot herbivores will provide us with new insights on the mechanisms of root-shoot communication. A systems-biological approach, whereby a detailed transcriptomic analysis of the systemic induced defense responses against nematodes and insects in roots and shoots is complemented with metabolic measurements, is necessary to obtain the most comprehensive view on the causes and consequences of double infestations. Surely, the model species *A. thaliana *could be used to investigate certain molecular mechanisms, but the small size and the short life cycle of this model plant makes it rather unsuitable to perform long-term investigations of the direct and indirect defense strategies of plants against these herbivores. Recent advances in genomic analysis of other relevant host plants such as *Brassica* spp., tomato, and potato should make this kind of studies feasible on a more realistic time scale.

Another issue to be resolved is the validity of lab-based experiments for processes in the fields. Therefore we suggest performing the laboratory experiments in such a way that they mimic ecological relevant conditions, including inoculum densities and developmental stage of the insects and plants at the time of infection. The results obtained under (semi)-controlled laboratory conditions should be complemented with field studies. This will not only indicate whether the mechanisms observed in the greenhouse are working under natural conditions, but it will also reveal the effect of additional factors influencing the performance of PPN, the foliar herbivore, and their hosts.

## Conflict of Interest Statement

The authors declare that the research was conducted in the absence of any commercial or financial relationships that could be construed as a potential conflict of interest.
